# Association between Family Dysfunction and Post-Traumatic Stress Disorder in School Students during the Second COVID-19 Epidemic Wave in Peru

**DOI:** 10.3390/ijerph19159343

**Published:** 2022-07-30

**Authors:** Maria A. Fernandez-Canani, Stefany C. Burga-Cachay, Mario J. Valladares-Garrido

**Affiliations:** 1 School of Medicine, Universidad de San Martín de Porres, Chiclayo 14012, Peru; maria_fernandez4@usmp.pe (M.A.F.-C.); stefany_burga@usmp.pe (S.C.B.-C.); 2 South American Center for Education and Research in Public Health, Universidad Norbert Wiener, Lima 15046, Peru; 3 Hospital Regional Lambayeque, Chiclayo 14012, Peru

**Keywords:** post-traumatic stress disorder, COVID-19, family dysfunction, Peru

## Abstract

Although the effect of the COVID-19 pandemic on children and adolescents’ mental health has been studied, there is still scarce evidence of the influence of nuclear family on the development of post-traumatic stress disorder (PTSD). This study aimed to determine the association between family dysfunction and PTSD in Peruvian high-school students during the COVID-19 pandemic. A cross-sectional study was conducted using a virtual survey administered to 562 high-school students in three schools in Chiclayo, Peru. The dependent variable was PTSD, which was measured with the Child PTSD Symptom Scale. Family dysfunction was the main independent variable, measured with the Family APGAR Questionnaire. Prevalence ratios (PR) and 95% confidence intervals (95% CI) were estimated with generalized linear models. Most of the students were female (88.3%) and the average age was 14.4 years. We found that 21.4% showed severe family dysfunction and 60.3% had PTSD. Students with mild and moderate family dysfunction had 37% (PR: 1.37; 95% CI: 1.14–1.65) and 26% (PR: 1.26; 95% CI: 1.04–1.54) higher PTSD prevalence, respectively. In conclusion, family dysfunction may influence the development of PTSD in adolescents. This study suggests the importance to develop a healthy family environment to help adolescents face critical situations experienced during the pandemic.

## 1. Introduction

Adolescence is a stage of biological, social, and mental changes [1]. Circumstances such as family influence, quality of life, and school performance, may generate psychological and emotional disorders such as depression, anxiety, and post-traumatic stress disorder (PTSD) [2].

A study conducted on American adolescents estimated a 6.3% prevalence of PTSD [3]. During the COVID-19 pandemic, a systematic review estimated that PTSD affects 48.0% of this population [4]. Some settings in this context have shown high rates of PTSD, such as Turkey (61.1%) [5] and Saudi Arabia (71.5%) [6].

Increasing rates of PTSD in adolescents are commonly attributed to natural disasters and wars. However, during the COVID-19 pandemic, PTSD has been linked to prolonged quarantine, fear of contagion, frustration, boredom, not seeing peers and teachers, lack of space at home, and the loss of loved ones [7].

The nuclear family successfully maintains the balance of its members [8] and represents a potential protective factor for the development of mental health disorders in children and adolescents [8]. Parents are the most immediate environment of adolescents, and the attachment between parents and their children is the engine of an adequate social integration and mental health [8]. However, single-parent families or divorce rates have increased in the last decades [9]. These examples of changes in family patterns are summed to maladaptive behaviors displayed by one or more family members, which determine an altered environment. This problem, known as family dysfunction, has led to traumatic events for children that increase the risk of PTSD [10,11].

To our knowledge, there are no studies during the COVID-19 context that have assessed the influence of family dysfunction on the development of PTSD in adolescents. Few pre-pandemic studies have evaluated this association: one in adolescents with cancer [11], and the other in adolescent victims of violence [10]. Similar studies have not measured other mental health outcomes (e.g., anxiety, depression, childhood trauma) [10,11], nor have measured variables potentially influencing PTSD (alcoholism, compliance with social isolation measures, previous history of mental health disorders, close relative with COVID-19, close relative deceased due to COVID-19, having sought mental health support during period of compulsory social isolation). In addition, studies that determined this association had a small sample size and presented measurement bias (not validated instruments) [10].

Therefore, we aimed to identify whether there is an association between family dysfunction and PTSD in adolescents in three schools in Peru, in the context of the COVID-19 pandemic.

## 2. Materials and Methods

### 2.1. Study Design

A cross-sectional study was conducted between March and April 2021 in secondary students in three schools in Chiclayo, Peru, in order to evaluate the association between family dysfunction and PTSD in the context of the COVID-19 pandemic. 

### 2.2. Population and Sample

**Figure 1:**
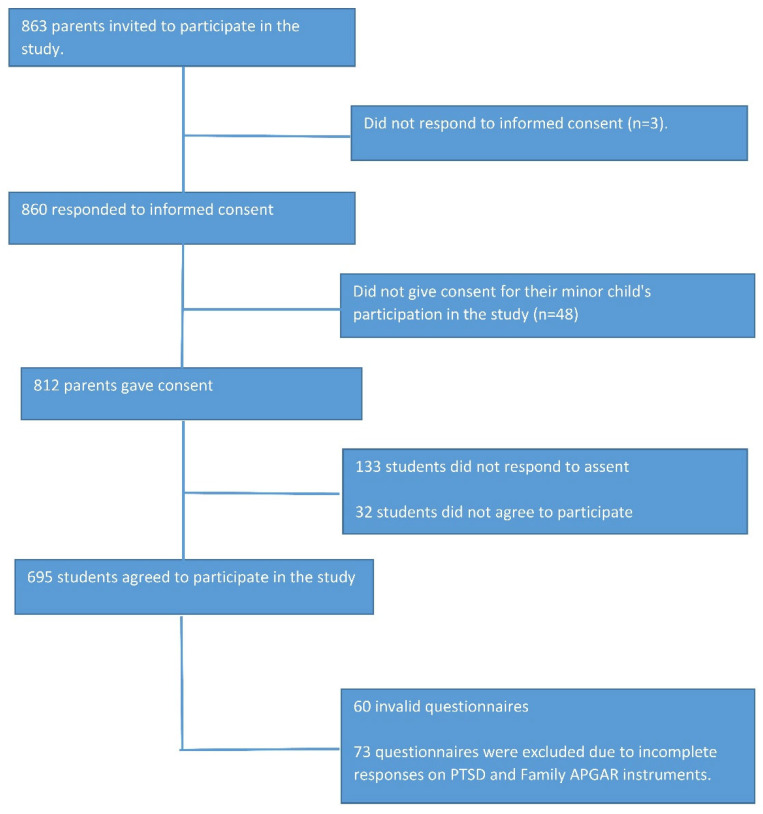
Flowchart of participant selection.

The study population consisted of 863 students enrolled in the 2021-II academic semester, comprising the following schools: IEP “Virgen Del Carmen” (N = 120 students), IEP “Trilce” (N = 293 students), and IEP “Inmaculada Concepción” (N = 450 students). We estimated a sample size based on a 43% and 57% prevalence of PTSD in unexposed and exposed individuals, respectively, a confidence level of 95%, a margin of error of 5%, and 80% power. Thus, a sample size of 400 participants was calculated. A 10% refusal rate of parents was added, as well as a 10% refusal rate for students, and a 10% rate of incomplete records. Finally, 520 participants were estimated for the study. Non-probability snowball sampling was performed. We included students aged between 11 and 18, whose parents and/or guardians voluntarily authorized informed consent, and schoolchildren who provided voluntary informed assent approval. We excluded 73 questionnaires from students who did not complete the instrument for the variables of interest of the research (Child PTSD Symptom Scale and Family APGAR). The final sample we collected for this study was 562 secondary school students from the three schools mentioned above (Figure 1).

### 2.3. Data Collection Procedures

For data collection, authorization was requested from the principals of the educational institutions. Subsequently, the purpose of the research was explained to the parents of the children in order to obtain their consent to carry out the study. An online survey was disseminated including all the instruments described in Section 2.4 and other relevant information mentioned in Section 2.5. Quality control was performed using a pilot pre-test in a group of 60 students with similar ages from the participant schools. We requested permission to access the school’s online platform in order to capture the desired information. The survey was conducted when the students were in virtual classes, in a period of no evaluations, during the break between classes, and with an approximate filling time of 15 min.

The data were collected and managed using the Research Electronic Data Capture system (REDCap). REDCap is a secure online platform for designing, managing, entering, and rigorously capturing surveys and online databases for research [12,13]. To design the online survey, a template was created in which all the data collection forms were to be included. We clicked on “Add new instrument” and created 3 instruments: (1) parent informed consent, (2) informed consent, and (3) data collection questionnaires; in this form, we designed all the questions for our study (General Data, Family Apgar Questionnaire, Child PTSD Symptom Scale, Connor–Davidson Resilience Scale 10, GAD-7 Scale, PHQ-9 Questionnaire, Marshall Scale for trauma, and Alcohol Use Disorders Identification Test). This process was performed within the Online Designer tool. 

Then, we used the Survey Queue tool. This tool allowed us to combine the list of all aforementioned questionnaires into one single form for each participant. To combine all questionnaires into this single form, we activated the Survey Queue in the REDCap project, we then navigated to “Online Designer”, and clicked on the Survey Queue icon located above the data collection instruments. Immediately, a “Set up Survey Queue” box appeared. Next, we clicked the Enable icon for each questionnaire we wanted to set up. Under the “Show survey in survey queue when...” column, we used the drop-down menu to indicate when each questionnaire should be shown to the participant. We used the Branching Logic tool in the Survey Queue to display the questions in the questionnaires. The Branching Logic tool allowed to display the questionnaires to the participants compiled on a single form automatically, only if the parents and adolescent provided informed consent and informed assent, respectively. 

In addition, we used other tools in the REDCap project to ensure the correct arrangement, provision, and completion of questionnaires: unique and anonymized identifiers on each form, questionnaires ordered in a consistent way (first consent and informed assent, general data, and then variables of interest instruments), use of conditional logic for skip questions, mandatory fields in questions to avoid missing, minimum, and maximum ranges in numerical variables, and use of groups matrix tool for Likert scale responses. Finally, a public survey link was created using the Manage Survey Participants tool. Before starting the pilot study and formal investigation, the survey link and form were verified to work correctly.

### 2.4. Instruments

**Family Apgar Questionnaire:** It is an instrument designed to evaluate family functionality. It consists of five items with a five-point Likert-type scale, where each item is scored on a frequency ranging from 0 (never), 1 (almost never), 2 (sometimes), 3 (almost always), and 4 (always). It is divided into five dimensions: adaptability, partnership, growth, affection, and resolve. The final value that the variable will adopt is as follows: good family function (18–20 points), mild family dysfunction (14–17 points), moderate family dysfunction (10–13 points), and severe family dysfunction (9 or fewer points). The correlation index is 0.80 [14]. Additionally, the Family Apgar was evaluated in multiple investigations, showing correlation indexes ranging from 0.71 to 0.83, for various realities [14]. In its original validation report in English, the APGAR showed adequate internal consistency (Cronbach’s alpha = 0.86) [15]. Subsequently, it was adapted to Spanish showing adequate internal consistency (α = 0.84) and, through exploratory factor analysis (EFA), it evidenced the presence of only one construct: family function [16]. Other versions available in Spanish are reported in Peru [17], and they show adequate internal consistency (α = 0.729 for Spanish in Peru). 

**Child PTSD Symptom Scale**: CPSS is a self-report instrument, with 17 items. It covers three symptom groups (re-experiencing, avoidance, and hyperarousal), equivalent to the 17 symptoms considered in the DSM-IV diagnostic criteria for PTSD. The response format is a four-point Likert scale, from 0 to 3 (0 = only at one time; 1 = once in a while; 2 = half the time; 3 = almost always). A score of 24 or more points allowed us to obtain a sensitivity of 82% and specificity of 88% regarding the diagnosis of PTSD with the DISC-IV [18]. The psychometric properties of this instrument translated into Spanish were evaluated in Chilean children and adolescents who were victims of sexual abuse, which yielded appropriate values of internal consistency, analogous to those of the original instrument (α = 0.92) [19]. 

**Connor–Davidson Resilience Scale 10:** It has ten items and uses a Likert-type scale from 0 (“not true at all”) to 4 (“true nearly all the time”) [20]. Total scores range from 0 to 40, the higher the score, the higher the level of resilience [20]. The instrument has good psychometric properties: internal consistency (Cronbach’s alpha) was α = 0.85 and construct validity [20]. In addition, in the Spanish-speaking population, it has good reliability and validity [21,22,23,24,25]. In an investigation, Cronbach’s alpha = 0.86, sensitivity = 70%, and specificity = 68.2% have been estimated [22].

**GAD-7 Scale (Generalized Anxiety Disorder-7):** It is a useful instrument to detect symptoms of generalized anxiety and their severity. It consists of seven items using a Likert scale from 0 to 3. Affective symptoms (fear, anxiety), cognitive symptoms (mental disintegration, apprehension), and somatic symptoms during the last two weeks are evaluated through this instrument. Spitzer et al. estimated a cut-off point of 10 points with 89% sensitivity and 82% specificity, and adequate construct validity [26]. It offers a total score from 0 to 21, which determines the severity of anxiety symptoms according to these intervals: no anxiety (0–4 points), mild anxiety (5–9 points), moderate anxiety (10–14 points), and severe anxiety (15–21 points) [27]. In the Hispanic American population, it was estimated to have adequate internal consistency (Cronbach’s alpha = 0.93) [28]. It has also been validated in Peru by Ventura et al., demonstrating adequate internal consistency (Cronbach’s alpha = 0.78) [27]. 

**Patient Health Questionnaire-9 (PHQ-9):** This instrument screens for depressive symptoms during the last two weeks. It consists of nine items, based on the nine criteria for the DSM-V diagnosis of major depression, scored with values of 0, 1, 2, and 3, according to the response categories corresponding to “not at all”, “several days”, “most days” and “almost every day”, respectively. The total score for the nine items ranges from 0 to 27. It has a validated version for the Peruvian population, using data from the Demographic and Family Health Survey (ENDES), where the validity and reliability (Cronbach’s alpha = 0.87) of the PHQ-9 questionnaire were optimal [29]. According to the score obtained, it is categorized into minimal depression (0–4 points), mild depression (5–9 points), moderate depression (10–14 points), moderate to severe depression (15–19 points), and severe depression (20–27 points) [30]. 

**Marshall Scale for trauma:** It is a questionnaire that assesses the presence or absence of traumatic events before 16 years of age through seven questions [31]. It has a maximum score of seven points. According to the score obtained, it is categorized into presence of trauma (1 or more points) and absence of trauma (0 points). In addition, the cut-off point to define polytrauma is three points or more [31]. It has been validated by Cuneo et al., who found optimal external validity (Pearson correlation coefficient: 0.88) [32]. In addition, it has been used and validated in studies in Latin America [33,34]. 

**Alcohol Use Disorders Identification Test (AUDIT):** The AUDIT test is a ten-question questionnaire with information on alcohol consumption [35,36]. It was originally developed by Saunders [35,36]. Subsequently, it was translated and validated in Spanish by Rubio et al. in primary care patients, and an optimal internal consistency was estimated (Cronbach’s alpha: 0.86), and 90% sensitivity and 90% specificity with a cut-off point of eight points were obtained [37]. It has three questions about the use of alcoholic beverages (quantity, frequency), four questions related to dependence, and the last three questions for the analysis of its consequences. The score range is from 0 to 40. It is considered positive if ≥eight points, for the detection of excessive alcohol consumption. In addition, it has low risk (0–7 points), medium risk (8–15 points), high risk (16–19 points), and probable addiction (20+ points) [38].

### 2.5. Variables

The dependent variable was PTSD, defined with the Child PTSD Symptom Scale when a score equal to or greater than 24 is obtained. The main independent variable was family dysfunction, defined as mild family dysfunction (14 to 17 points), moderate family dysfunction (10 to 13 points), and severe family dysfunction (9 or fewer points), assessed with the APGAR questionnaire. The secondary independent variables were results from the instruments of resilience, depression, anxiety, childhood trauma, and alcohol consumption, as well as self-reported information on socio-educational features (gender, age, school year), compliance with isolation measures (no, yes), perception of severity of the COVID-19 pandemic (very serious, serious, neutral, overrated, very overrated), confidence in the government to manage the COVID-19 epidemic (much trust, little trust, not trust nor distrust, little distrust, much distrust), having a family member with recent COVID-19 (no, yes), having a family member deceased from COVID-19 (no, yes), and presenting a previous history of mental health disorders (no, yes).

### 2.6. Data Analysis

We exported the Excel database from the REDcap system. Subsequently, we proceeded to report the absolute and relative frequencies of the categorical variables. We calculated the measures of central tendency (mean) and measures of dispersion (standard deviation) of the numerical variables.

Through bivariate analysis, we evaluated the assumption of expected frequencies using the chi-square test to compare proportions. In the case of numerical variables (age and resilience), the Student’s *t*-test was used, after the evaluation of the normal distribution assumption. A significance level of 5% was used. 

In the simple and multiple regression analysis, prevalence ratios (PR) and 95% confidence intervals were estimated using generalized linear models (GLM), Poisson distribution family, and log link function with robust variance. Using simple regression analysis, the association between PTSD and family dysfunction was evaluated as well as the other covariates of interest. In multiple regression, we controlled for the association of interest (family dysfunction and PTSD) with the confounding variables. We assessed the collinearity of the confounding variables included in the adjusted model. Stata v.17.0 statistical software was used.

### 2.7. Ethical Aspects

The present research was approved by the Ethics and Research Committee of Universidad San Martín de Porres (N° 370-2021-CIEI-FMH-USMP). During data collection, the parents of the minors surveyed were informed of the objectives of the research, as well as the benefits that would be obtained by conducting this study. Only adolescents whose parents accepted their participation by means of informed consent were included. In addition, minors also agreed to participate through informed assent. During data collection, processing, and analysis, the confidentiality of the adolescents was respected, and we committed not to divulge their personal information.

## 3. Results

Out of 562 students, we found that the mean age was 14.41, the majority were female (88%) and were in the fifth year of secondary school (35.4%). Of the total, 48.6% reported having had a deceased relative due to COVID-19 and 18.0% had sought mental health support. The majority had mild depression (30.0%) and mild anxiety (27.9%). Of the total, 42.5% had childhood trauma and 60.3% had post-traumatic stress disorder. Regarding family dysfunction, 21.4% had it (Table 1).

In Table 2, we found that the prevalence of post-traumatic stress in students with severe family dysfunction is 38.7% higher compared to those who did not have alteration in regard to Family APGAR. A higher proportion of post-traumatic stress was observed in schoolchildren with moderate-severe depression, compared to those without depression (92.3% vs. 18.8%; *p* < 0.001). Students with severe anxiety had a 69.4% higher frequency of post-traumatic stress, compared to those who were not anxious (98.5% vs. 29.1%; *p* < 0.001). Additionally, factors associated with having post-traumatic stress were age (*p* < 0.001), gender (*p* < 0.001), school year (*p* < 0.001), having confidence in the government to manage the COVID-19 pandemic (*p* < 0.001), history of mental health (*p* < 0.001), having sought mental health support (*p* < 0.001), resilience (*p* < 0.001), and childhood trauma (*p* < 0.001).

In the simple regression analysis, adjusted for the covariates of interest, we observed that the prevalence of post-traumatic stress increases 86% (PR = 1.86; 95% CI: 1.51–2.30), 108% (PR = 2.08; 95% CI: 1.69–2.56), and 105% (PR = 2.05; 95% CI: 1.67–2.51) in students with mild, moderate, and severe family dysfunction, respectively. This is similar to what was observed in multiple regression, except for severe family dysfunction. Students with mild and moderate family dysfunction had a 37% (PR = 1.37; 95% CI: 1.14–1.65) and 26% (PR = 1.26; 95% CI: 1.04–1.54) higher prevalence of post-traumatic stress, with respect to relative to students without family dysfunction. Moreover, we found that students who sought mental health support had a 20% higher prevalence of PTSD (PR = 1.20; 95% CI: 1.06–1.35). The higher the level of depression, the higher the prevalence of PTSD, as students with major depression had a 162% higher prevalence of PTSD (PR = 2.62; 95% CI: 1.67–4.09). Students with mild anxiety (PR = 1.68; 95% CI: 1.29–2.18), moderate anxiety (PR = 1.62; 95% CI: 1.23–2.12), and severe anxiety (PR = 1.71; 95% CI: 1.31–2.24) were associated with a higher prevalence of PTSD (Table 3).

## 4. Discussion

### 4.1. Prevalence of Post-Traumatic Stress

We found that six out of ten students experienced post-traumatic stress (60.3%). This is similar to what was reported in studies conducted on adolescents in Turkey where the frequency of PTSD was 61.1% during the COVID-19 context [5]. Additionally, in Saudi Arabia, a study conducted on children and adolescents showed that 71.5% of the participants had PTSD symptoms during the COVID-19 quarantine [6]. However, our finding is contrary to a study conducted in France during the COVID-19 pandemic, in which 19.5% of students had probable PTSD [39]. Moreover, in China, 10.4% of adolescents were found to have PTSD during the pandemic [40]. The prevalence found in this study could be explained by traumatic events related to the pandemic, such as isolation, lifestyle disruption, and death of a family member. The COVID-19 outbreak has generated closures of playgrounds, schools, recreational areas, and beaches. That, in addition to character, age, and underlying health conditions of adolescents, predispose them to a higher risk of post-traumatic stress. Therefore, it is important to understand the psychological disruption that the pandemic may have generated in adolescents. Families and schools play a fundamental role, so consideration should be given to establishing a community support network, especially identifying children and adolescents at higher risk of mental disorders.

### 4.2. Family Dysfunction and Post-Traumatic Stress

This study showed that students with mild and moderate family dysfunction had 37% and 26% higher frequency of PTSD, respectively. We have not identified studies evaluating the association of interest in the context of COVID-19. However, a study in China concluded that healthy family function can alleviate generalized anxiety disorder in college students during the COVID-19 pandemic [41]. In the pre-pandemic period, a study evaluating PTSD in the Mexican pediatric population found that family integration or not would not determine the development of PTSD [42]. Another study conducted on U.S. adolescents with cancer showed a higher frequency of PTSD in those with poor family functioning [11]. The association found here could be explained by the fact that an unhealthy family environment may worsen the effects generated by stressful and traumatic events in adolescents. For example, single-parent families, divorce, family violence, abandonment, or authoritarianism can generate emotional distancing and a distressing relationship with parents. These ongoing problems in children’s lives can lead to PTSD and other mental disorders [43]. In addition, the negative effect of family dysfunction may be increased by the COVID-19 pandemic due to isolation, less physical interaction with friends, fear of contagion, but also the sense of distress generated by the unhealthy interaction within the dysfunctional family [44]. To resolve this unfortunate situation, communication and emotional exchange have been shown to be key factors that improve children’s mental well-being [45]. Therefore, it is important to highlight that the mental state of parents and their ability to convey security to children reduces psychological distress [45,46]. In this sense, the emotional support of parents could be ensured by promoting community strategies that help parents or caregivers to preserve mental health, especially in difficult situations such as the COVID-19 pandemic [47].

### 4.3. Other Factors Associated with Post-Traumatic Stress

It was found that students with depression had a higher frequency of PTSD, and this gradually increased as depressive symptoms became more pronounced. In China, there was a correlation between PTSD and depressive symptoms in 10.7% of adolescents. Earthquake-related exposure, negative life events, previous exposure to the Wenchuan (China) earthquake in 2008, and being left behind by parents contributed to PTSD and depressive symptoms [48]. No literature contrary to this association was found. The association found could be explained because of monotony, disappointment, lack of face-to-face contact with classmates, friends, and teachers, economic losses, changes in daily routine, home confinement, death of a family member from COVID-19, and fear of infection could originate or intensify mental problems [49]. 

We found a higher prevalence of PTSD in adolescents with mild and moderate anxiety. This is similar to the study carried out in Ecuador where it was observed that there is 28.6 times more probability of having PTSD with anxiety disorder compared to those who do not have this disorder (11). In Indonesia, it was found that the associations and comorbidity between PTSD, depression, and anxiety were statistically significant (*p* = 0.001) [50]. There was no evidence of the contrary. The association found may be due to multiple factors (fear of contagion, frustration, boredom, inadequate information, lack of in-person contact with classmates, friends, and teachers, lack of personal space at home, and economic problems at home). These conditions would cause anxious symptoms, which, added to pre-existing anxiety in adolescents, produce psychological vulnerability, leading to an increased risk of having PTSD (51).

Adolescents with higher levels of resilience had a lower frequency of PTSD. This is similar to what was reported in Wuhan, China, in students affected by the COVID-19 pandemic, where resilience positively influenced the mental health of students [51]. This is contrary to a study conducted in Brazil in which no association was found between resilience and PTSD [52]. There were no studies that proved contrary to what was found in our research. This association could be due to the fact that confinement may have favored the development of family resilience factors such as spending more time with parents [53].

Another association observed was that adolescents who sought psychological support had a higher prevalence of PTSD. This differs from a study by Wethington HR. et al., who showed evidence that individual and group cognitive-behavioral therapy helps attenuate psychological harm in children and adolescents with PTSD symptoms exposed to trauma [54]. This association is due to the fact that psychotherapy puts the adolescent’s traumatic experiences in a symbolic order, taking him/her to a time and space different from the present, allowing him/her to recall the traumatic event without reliving it.

### 4.4. Implications of Findings in Mental Health Policy

Our findings provide evidence that family dysfunction is a negative indicator of mental health in adolescents. In addition to this problem, the COVID-19 pandemic has added detrimental effects related to mitigation strategies, such as prolonged school closures and home confinement. Measures to manage the increased rates of PTSD, as well as other psychological problems, have been hampered by limited access to mental health services [55]. Therefore, family dysfunction must be recognized by society, authorities, and organizations as a critical problem in the lives of adolescents. In this context, the reduction in dysfunctional families should be considered a goal in development strategies.

### 4.5. Limitations and Strengths

This study has some limitations. First, measurement bias is one of them since it is not possible to infer the results to the entire secondary school population, as it was only possible to select three educational institutions in this study. Second, information bias is another limitation. Because of the state of health emergency caused by COVID-19, the surveys were conducted virtually and not all students had access to the Internet or did not answer all the questions in the surveys. Third, due to the cross-sectional design of the study, causality cannot be attributed between the variables of interest due to the absence of temporality between the information collected.

However, the main strength of this research lies in the fact that it has been possible to capture a broad and diverse sample of secondary students from Lambayeque, a region severely hit by the COVID-19 pandemic, during the period of social distancing. Additionally, the large sample obtained allowed us to obtain accurate results and provide a great approximation to understand the potential relationship between family dysfunction and the presence of PTSD, as well as other influential factors of this mental health disorder. Finally, the variables of interest were measured through instruments with adequate psychometric properties.

## 5. Conclusions

Adolescents who experience family dysfunction have a higher prevalence of PTSD. Our finding reaffirms the importance of developing a healthy family environment, as this helps adolescents to cope with critical situations experienced in the pandemic.

## Figures and Tables

**Table 1:** Characteristics of participants (*n* = 562).

Characteristics	*n* (%)
Age (years) *	14.41 ± 1.41
**Gender**	
Male	66 (11.7)
Female	496 (88.3)
**School year**	
First	83 (14.8)
Second	63 (11.2)
Third	71 (12.6)
Fourth	146 (26.0)
Fifth	199 (35.4)
**Compliance with isolation measures**	
No	24 (4.3)
Yes	538 (95.7)
**COVID-19 pandemic severity degree**	
Very serious	403 (71.7)
Serious	109 (19.4)
Neutral	27 (4.8)
Overrated	10 (1.8)
Very overrated	13 (2.3)
**Trust in the government to manage the COVID-19 pandemic**	
Much trust	18 (3.2)
Little trust	155 (27.6)
Not trust, nor distrust	178 (31.7)
Little distrust	119 (21.2)
Much distrust	92 (16.4)
**Family member with recent COVID-19**	
No	136 (24.2)
Yes	426 (75.8)
**Family member deceased due to COVID-19**	
No	289 (51.4)
Yes	273 (48.6)
**Previous history of mental health disorders**	
No	493 (87.7)
Yes	69 (12.3)
**Seeking mental health support ^†^**	
No	461 (82.0)
Yes	101 (18.0)
**Mental health support source**	
Family	37 (36.6)
School	2 (2.0)
MINSA mental health facility	13 (12.9)
Others	49 (48.5)
**Depression ^†^**	
Minimal	112 (23.6)
Mild	142 (30.0)
Moderate	93 (19.6)
Moderate serious	65 (13.7)
Serious	63 (13.3)
**Anxiety ^†^**	
No	175 (37.8)
Mild	129 (27.9)
Moderate	91 (19.7)
Severe	68 (14.7)
**Resilience**	24.32 ± 8.09
**Alcohol consumption**	
Low risk	458 (92.9)
Medium risk	29 (5.9)
High risk	4 (0.8)
Probable addiction	2 (0.4)
**Childhood trauma ^†^**	
No	263 (57.6)
Yes	194 (42.5)
**Family dysfunction**	
No	205 (36.5)
Mild	136 (24.2)
Moderate	101 (18.0)
Severe	120 (21.4)
**Post-traumatic stress**	
No	223 (39.7)
Yes	339 (60.3)

^†^ The sum of the variables is not 562 due to missing values. * Mean and standard deviation.

**Table 2:** Factors associated with post-traumatic stress.

Variables	Post-Traumatic Stress		*p* *
	No (*n* = 223)	Yes (*n* = 339)	
	*n* (%)	*n* (%)	
Age (years) **	14.16 ± 1.52	14.59 ± 1.31	0.001
**Gender**			<0.001
Male	42 (63.6)	24 (36.4)	
Female	181 (36.5)	315 (63.5)	
**School grade**			0.001
First	47 (56.6)	36 (43.4)	
Second	31 (49.2)	32 (50.8)	
Third	28 (39.4)	43 (60.6)	
Fourth	46 (31.5)	100 (68.5)	
Fifth	71 (35.7)	128 (64.3)	
**Compliance with isolation measures**			0.823
No	9 (37.5)	15 (62.5)	
Yes	214 (39.8)	324 (60.2)	
**COVID-19 pandemic severity degree**			0.562
Very serious	159 (39.5)	244 (60.6)	
Serious	43 (39.5)	66 (60.6)	
Neutral	12 (44.4)	15 (55.6)	
Overrated	2 (20.0)	8 (80.0)	
Really overrated	7 (53.9)	6 (46.2)	
**Trust in the government to manage the COVID-19 pandemic**			<0.001
Much trust	12 (66.7)	6 (33.3)	
Little trust	79 (51.0)	76 (49.0)	
Not trust, nor distrust	55 (30.9)	123 (69.1)	
Little distrust	43 (36.1)	76 (63.9)	
Much distrust	34 (37.0)	58 (63.0)	
**Family member who have suffered from COVID-19**			0.224
No	60 (44.1)	76 (55.9)	
Yes	163 (38.3)	263 (61.7)	
**Family member deceased due to COVID-19**			0.907
No	114 (39.5)	175 (60.6)	
Yes	109 (39.9)	164 (60.1)	
**Previous history of mental health disorder**			<0.001
No	219 (44.4)	274 (55.6)	
Yes	4 (5.8)	65 (94.2)	
**Mental health support search**			<0.001
No	205 (44.5)	256 (55.5)	
Yes	18 (17.9)	83 (82.2)	
**Depression**			<0.001
Minimal	91 (81.3)	21 (18.8)	
Mild	59 (41.6)	83 (58.5)	
Moderate	21 (22.6)	72 (77.4)	
Moderate to serious	5 (7.7)	60 (92.3)	
Serious	0 (0.0)	63 (100)	
**Anxiety**			<0.001
No	124 (70.9)	51 (29.1)	
Mild	34 (26.4)	95 (73.6)	
Moderate	11 (12.1)	80 (87.9)	
Severe	1 (1.5)	67 (98.5)	
Resilience **	26.99 ± 8.26	22.78 ±7.58	<0.001
**Alcohol**			0.179
Low risk	175 (38.2)	283 (61.8)	
Medium risk	8 (27.6)	21 (72.4)	
High risk	0 (0.0)	4 (100.0)	
Probable addiction	0 (0.0)	2 (100.0)	
**Childhood trauma**			<0.001
No	124 (47.2)	139 (52.9)	
Yes	42 (21.7)	152 (78.4)	
**Family APGAR**			<0.001
Normal	129 (62.9)	76 (37.1)	
Mild	42 (30.9)	94 (62.1)	
Moderate	23 (22.8)	78 (77.2)	
Severe	29 (24.2)	91 (75.8)	

* *p* value calculated with the chi-square of independence. ** Mean and standard deviation. *p* value calculated with the Student’s *t* test.

**Table 3:** Factors associated with post-traumatic stress disorder in students at three schools in Chiclayo, 2021, simple and multiple regression analysis.

Characteristics	Post-Traumatic Stress					
	Simple Regression			Multiple Regression *		
	PR	95% CI	*p* **	PR	95% CI	*p* **
**Age (years)**	1.09	1.04–1.15	0.001	1.00	0.87–1.14	0.948
**Gender**						
Male	Ref.			Ref.		
Female	1.75	1.26–2.42	0.001	1.33	0.98–1.80	0.065
**School grade**						
First	Ref.			Ref.		
Second	1.17	0.83–1.66	0.371	0.89	0.65–1.21	0.454
Third	1.40	1.02–1.90	0.035	0.78	0.54–1.13	0.196
Fourth	1.58	1.21–2.07	0.001	0.88	0.57- 1.36	0.567
Fifth	1.48	1.14–1.94	0.004	0.91	0.53–1.57	0.738
**Compliance with isolation measures**						
No	Ref.			Ref.		
Yes	0.96	0.70–1.32	0.819	1.01	0.80–1.27	0.949
**COVID-19 pandemic severity degree**						
Very serious	Ref.			Ref.		
Serious	1.00	0.84–1.19	0.999	1.00	0.88–1.14	0.998
Neutral	0.92	0.65–1.30	0.627	1.12	0.76–1.64	0.570
Overrated	1.32	0.96–1.82	0.088	1.10	0.77–1.56	0.614
Really overrated	0.76	0.42–1.38	0.370	0.66	0.35–1.24	0.197
**Trust in the government management**						
Much trust	Ref.			Ref.		
Little trust	1.47	0.75–2.88	0.261	1.36	0.74–2.48	0.321
Not trust, nor distrust	2.07	1.07–4.02	0.031	1.59	0.88–2.89	0.126
Little trust	1.92	0.98–3.74	0.056	1.65	0.90–3.02	0.102
Much distrust	1.89	0.97–3.70	0.063	1.55	0.84–2.87	0.159
**Family member with recent COVID-19**						
No	Ref.			Ref.		
Yes	1.10	0.93–1.31	0.243	0.98	0.85–1.22	0.731
**Close relative deceased due to COVID-19**						
No	Ref.			Ref.		
Yes	0.99	0.87–1.13	0.907	1.01	0.90–1.13	0.892
**Previous history of mental health disorders**						
No	Ref.			Ref.		
Yes	1.69	1.53–1.87	<0.001	0.98	0.87–1.10	0.711
**Seeking mental health support**						
No	Ref.			Ref.		
Yes	1.48	1.31 – 1.67	<0.001	1.20	1.06 – 1.35	**0.003**
**Depression**						
Minimal	Ref.			Ref.		
Mild	3.12	2.07–4.70	<0.001	2.11	1.37–3.24	**0.001**
Moderate	4.13	2.76–6.17	<0.001	2.44	1.57–3.79	**<0.001**
Moderate to serious	4.92	3.33–7.29	<0.001	2.57	1.66–4.00	**<0.001**
Serious	5.33	3.63–7.85	<0.001	2.62	1.67–4.09	**<0.001**
**Anxiety**						
No	Ref.			Ref.		
Mild	2.53	1.96–3.26	<0.001	1.68	1.29–2.18	**<0.001**
Moderate	3.02	2.36–3.85	<0.001	1.62	1.23–2.12	**0.001**
Severe	3.38	2.68–4.27	<0.001	1.71	1.31–2.24	**<0.001**
**Resilience**	0.98	0.97–0.99	<0.001	0.99	0.98–1.00	**0.006**
**Alcohol**						
Low risk	Ref.			Ref.		
Medium risk	1.71	0.93–1.48	0.188	0.97	0.78–1.21	0.809
High risk	1.62	1.51–1.74	<0.001	1.11	0.75–1.66	0.603
Probable addiction	1.62	1.51–1.74	<0.001	1.51	0.91–2.53	0.114
**Childhood trauma**						
No	Ref.			Ref.		
Yes	1.48	1.29–1.70	<0.001	1.04	0.93–1.17	0.479
**Family APGAR**						
Normal	Ref.			Ref.		
Mild	1.86	1.51–2.30	<0.001	1.37	1.14–1.65	0.001
Moderate	2.08	1.69–2.56	<0.001	1.26	1.04–1.54	0.021
Severe	2.05	1.67–2.51	<0.001	1.08	0.89–1.31	0.461

* Adjusted for covariates of interest. ** *p* values obtained with generalized linear models (GLM), Poisson family, log-link function, robust variance.

## Data Availability

The dataset generated and analyzed during the current study is not publicly available because the ethics committee has not provided permission/authorization to publicly share the data but are available from the corresponding author on reasonable request.
